# Supplementary material for: Reductions in commuting mobility correlate with geographic differences in SARS-CoV-2 prevalence in New York City
Source: Nat Commun. 2020 Sep 16;11:4674. doi: 10.1038/s41467-020-18271-5 (PMC7494926; doi:10.1038/s41467-020-18271-5)
Supplement: Supplementary file 1 — Supplementary Information [file 41467_2020_18271_MOESM1_ESM.pdf]

Supplementary Information for “Reductions in commuting mobility correlate with geographic differences in SARS-CoV-2 prevalence in New York City” by Kissler *et al.*

Borough	ZIP3
Bronx	104
Brooklyn	112
Manhattan	100, 101, 102
South Queens	110, 114, 116
North Queens	111, 113
Staten Island	103

Supplementary Table 1. 3-digit ZIP codes corresponding to the New York City boroughs. Source data are provided as a Source Data file.

Borough	Week	Number of tests	Number positive	Percent positive
Bronx	16 Mar	8	1	12.5
	23 Mar	60	12	20
	30 Mar	89	19	21.3
	6 Apr	66	11	16.7
	13 Apr	42	9	21.4
	20 Apr	16	0	0
	27 Apr	28	5	17.9
Brooklyn	16 Mar	3	0	0
	23 Mar	77	13	16.9
	30 Mar	85	18	21.2
	6 Apr	81	14	17.3
	13 Apr	79	11	13.9
	20 Apr	31	5	16.1
	27 Apr	30	3	10
Manhattan	16 Mar	14	1	7.1
	23 Mar	139	11	7.9
	30 Mar	173	21	12.1
	6 Apr	162	20	12.3
	13 Apr	138	13	9.4
	20 Apr	44	3	6.8
	27 Apr	48	3	6.2
North Queens	16 Mar	-	-	-
	23 Mar	59	9	15.3
	30 Mar	78	13	16.7
	6 Apr	62	8	12.9
	13 Apr	46	3	6.5
	20 Apr	19	3	15.8
	27 Apr	11	2	18.2
South Queens	16 Mar	-	-	-
	23 Mar	15	3	20
	30 Mar	17	5	29.4
	6 Apr	12	2	16.7
	13 Apr	8	1	12.5
	20 Apr	3	1	33.3
	27 Apr	3	1	33.3
Overall	16 Mar	25	2	8
	23 Mar	350	48	13.7
	30 Mar	442	76	17.2
	6 Apr	383	55	14.4
	13 Apr	313	37	11.8
	20 Apr	113	12	10.6
	27 Apr	120	14	11.7

Supplementary Table 2. Weekly number of SARS-CoV-2 tests (overall and positive) by borough. Source data are provided as a Source Data file.

Borough	70% sensitivity	80% sensitivity	90% sensitivity
Bronx	26.6 (20.8, 33.0)	23.4 (18.1, 29.1)	20.8 (16.2, 25.7)
Brooklyn	23.9 (18.8, 29.5)	20.9 (16.4, 25.7)	18.6 (14.7, 23.0)
Manhattan	14.5 (11.5, 17.8)	12.7 (10.1, 15.6)	11.3 (8.9, 13.9)
North Queens	20.1 (14.7, 26.3)	17.6 (12.8, 22.9)	15.7 (11.4, 20.5)
South Queens	33.4 (19.5, 49.7)	29.2 (17.0, 43.7)	26.0 (15.3, 38.9)
Overall	20.0 (17.8, 22.4)	17.5 (15.5, 19.6)	15.6 (13.9, 17.4)

Supplementary Table 3. Mean posterior percent population prevalence with 95% credible interval of SARS-CoV-2 infection by borough. The test is assumed to have perfect specificity and either 70%, 80%, or 90% sensitivity. Source data are provided as a Source Data file.

Borough	Percent decline
Bronx	49.5
Brooklyn	52.4
Manhattan	68.7
North Queens	57.4
South Queens	41.4

Supplementary Table 4. Changes in mobility by borough. The ‘percent decline’ column captures the reduction in transitions out of and into of each borough in the morning/evening during the study period compared to the 45-day period preceding February 26th, 2020. Aggregated morning trips out of the boroughs and evening trips into the boroughs here act a proxy for commuting to/from work. Source data are provided as a Source Data file.

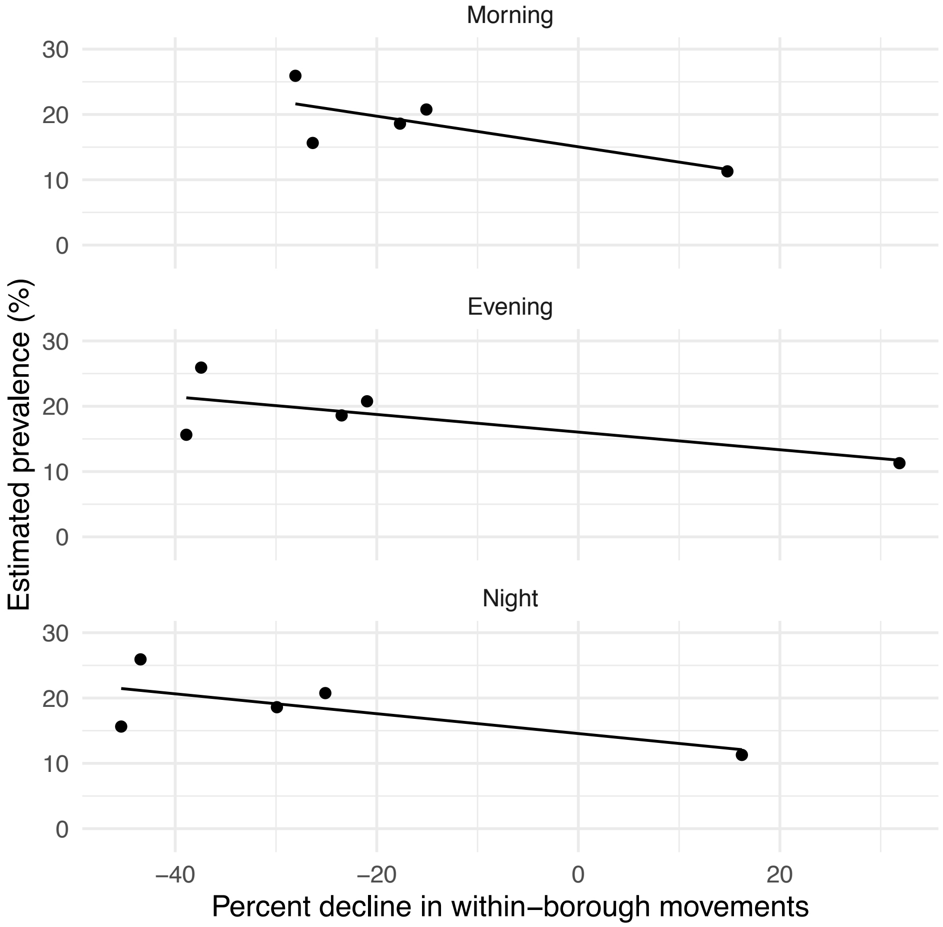

Supplementary Figure 1. Relationship between changes in within-borough movements and estimated prevalence. These scatterplots depict the relationship between the estimated posterior prevalence of SARS-CoV-2, assuming a 90% test sensitivity, and the percent decline in the number of within-borough movements during the study period *vs*. the 45 days preceding Feb 26th, 2020. Negative values on the horizontal axis indicate that the number of within-borough movements increased during the study period relative to the pre-pandemic control. The trends are all negative but non-significant according to a two-sided *t*-test for the slope (morning: p=0.16; evening: p=0.18; night: p=0.20).

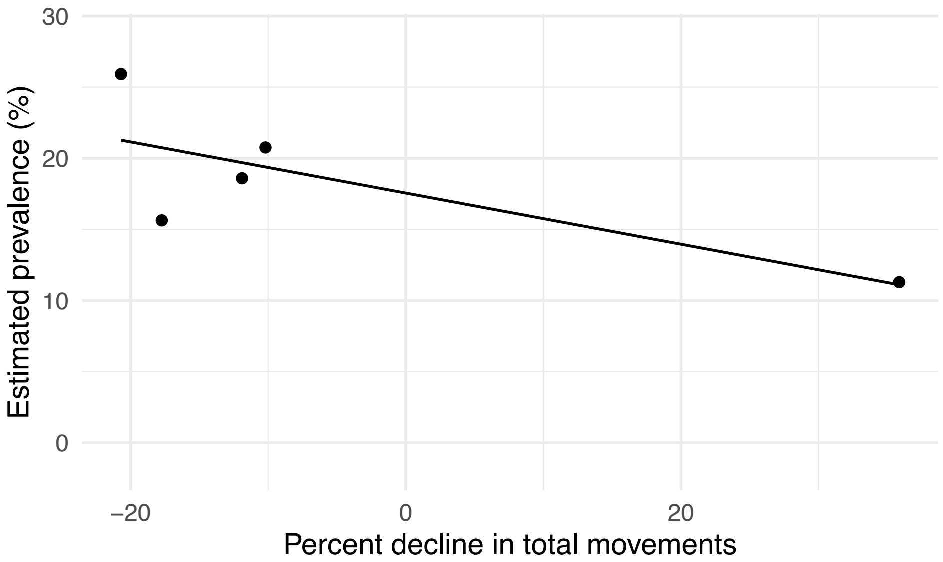

Supplementary Figure 2. Relationship between total change in movement and estimated prevalence. This scatterplot depicts the posterior prevalence of SARS-CoV-2 assuming a 90% test sensitivity as a function of the percent decline in total movements for each borough during the study period compared to the 45 days preceding Feb 26th, 2020. Negative values on the horizontal axis indicate that the total number of movements increased for the borough during the study period relative to the pre-pandemic control. The trend is negative but non-significant according to a two-sided *t*-test for the slope ($p = 0.14$).

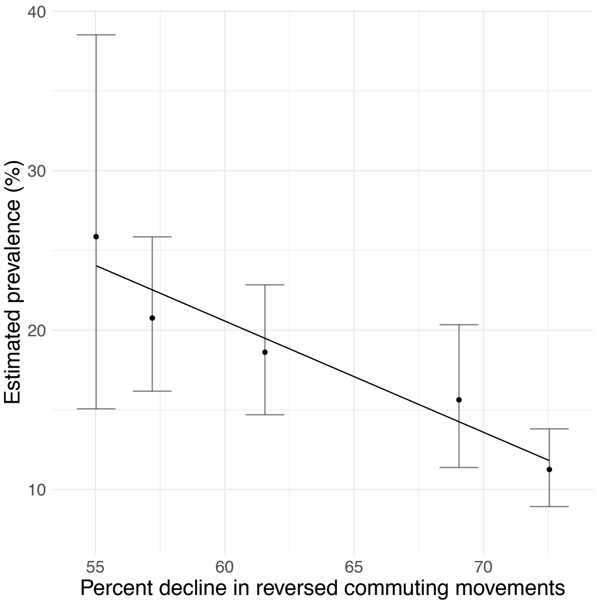

Supplementary Figure 3. Relationship between reverse commuting movements and estimated prevalence. This scatterplot depicts the posterior prevalence of SARS-CoV-2 assuming a 90% test sensitivity as a function of the percent decline in total morning movements into each borough and evening movements out of each borough during the study period compared to the 45 days preceding Feb 26th, 2020. These movements represent the reverse of the commuting-style movements considered in the main analysis. Error bars represent 95% credible intervals. There is a strong negative relationship (Pearson R = –0.86, [–0.55, -0.99]) indicating that greater declines in reverse commuting movements are also associated with lower prevalence.

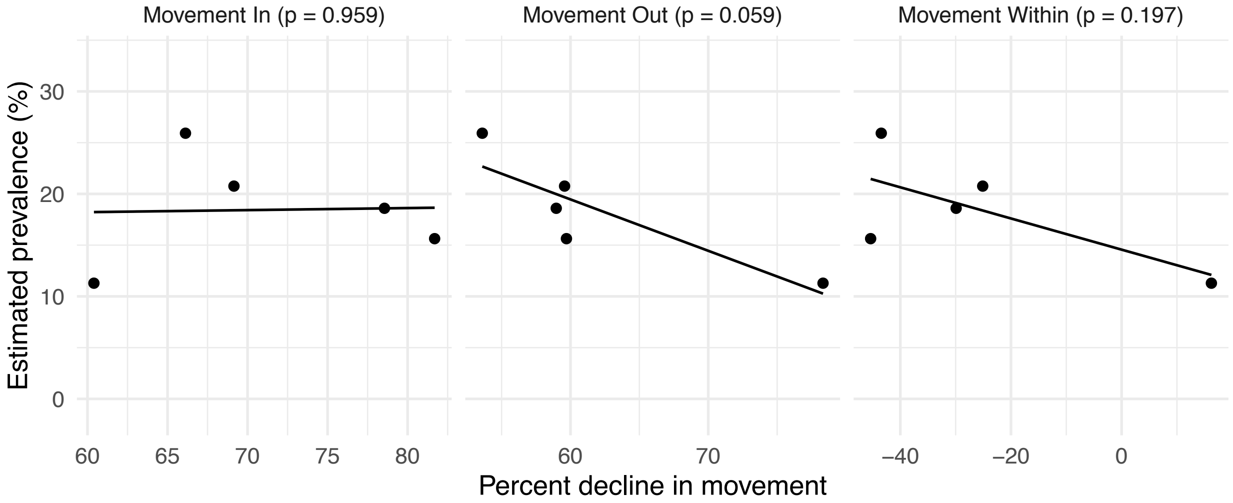

Supplementary Figure 4. Relationship between nighttime changes in movement and estimated prevalence. These scatterplots depict the posterior prevalence of SARS-CoV-2 assuming a 90% test sensitivity as a function of the percent decline in nighttime movements into, out of, and within each borough during the study period compared to the 45 days preceding Feb 26th, 2020. Negative values on the horizontal axis indicate that the total number of movements increased for the borough during the study period relative to the pre-pandemic control. While there is a negative relationship between the decline in nighttime movements out and nighttime movements within boroughs and the estimated prevalence of SARS-CoV-2, none of the relationships are significant at the $p = 0.05$ level according to a two-sided t-test for the slope.
