# Supplementary material for: Reductions in commuting mobility correlate with geographic differences in SARS-CoV-2 prevalence in New York City
Source: Nat Commun. 2020 Sep 16;11:4674. doi: 10.1038/s41467-020-18271-5 (PMC7494926; doi:10.1038/s41467-020-18271-5)
Supplement: Supplementary file 3 — Reporting Summary [file 41467_2020_18271_MOESM3_ESM.pdf]

nature research

Corresponding author(s): Yonatan Grad, Stephen Kissler

Last updated by author(s): 6 Aug 2020

Reporting Summary

Nature Research wishes to improve the reproducibility of the work that we publish. This form provides structure for consistency and transparency in reporting. For further information on Nature Research policies, see our Editorial Policies and the Editorial Policy Checklist.

Statistics

For all statistical analyses, confirm that the following items are present in the figure legend, table legend, main text, or Methods section.

n/a	Confirmed	
☐	☒	The exact sample size (*n*) for each experimental group/condition, given as a discrete number and unit of measurement
☐	☒	A statement on whether measurements were taken from distinct samples or whether the same sample was measured repeatedly
☐	☒	The statistical test(s) used AND whether they are one- or two-sided<br>*Only common tests should be described solely by name; describe more complex techniques in the Methods section.*
☐	☒	A description of all covariates tested
☐	☒	A description of any assumptions or corrections, such as tests of normality and adjustment for multiple comparisons
☐	☒	A full description of the statistical parameters including central tendency (e.g. means) or other basic estimates (e.g. regression coefficient) AND variation (e.g. standard deviation) or associated estimates of uncertainty (e.g. confidence intervals)
☐	☒	For null hypothesis testing, the test statistic (e.g. *F*, *t*, *r*) with confidence intervals, effect sizes, degrees of freedom and *P* value noted<br>*Give P values as exact values whenever suitable.*
☐	☒	For Bayesian analysis, information on the choice of priors and Markov chain Monte Carlo settings
☐	☒	For hierarchical and complex designs, identification of the appropriate level for tests and full reporting of outcomes
☐	☒	Estimates of effect sizes (e.g. Cohen's *d*, Pearson's *r*), indicating how they were calculated

Our web collection on statistics for biologists contains articles on many of the points above.

Software and code

Policy information about availability of computer code

Data collection	Data was collected on computers using Microsoft Excel.
Data analysis	Data analysis was conducted in R Version 3.6.2. All code is available at https://github.com/gradlab/COVID_NYC

For manuscripts utilizing custom algorithms or software that are central to the research but not yet described in published literature, software must be made available to editors and reviewers. We strongly encourage code deposition in a community repository (e.g. GitHub). See the Nature Research guidelines for submitting code & software for further information.

Data

Policy information about availability of data

All manuscripts must include a data availability statement. This statement should provide the following information, where applicable:

- Accession codes, unique identifiers, or web links for publicly available datasets
- A list of figures that have associated raw data
- A description of any restrictions on data availability

Data are available at https://github.com/gradlab/COVID_NYC

Field-specific reporting

Ecological, evolutionary & environmental sciences study design

All studies must disclose on these points even when the disclosure is negative.

Study description	Observational study of universal SARS-CoV-2 test results obtained from women hospitalized for delivery
Research sample	2,011 women with New York City ZIP codes hospitalized for delivery.
Sampling strategy	We analyzed SARS-CoV-2 test results administered universally to 2,011 pregnant women admitted for delivery at four NewYork Presbyterian hospital campuses (Columbia University Irving Medical Center/NYP-CUIMC, Weill Cornell Medical Center/NYP-WCM, Lower Manhattan Hospital/NYP-LMH, and Queens Hospital/NYP-Queens), Mount Sinai Hospital (MSH), and Mount Sinai West (MSW) hospital between March 22nd and May 3rd, 2020. NYP-CUIMC tests included those from NYP-Morgan Stanley Children's Hospital and NYP-Allen Hospital. Rationale: The timing of the study coincided with peak SARS-CoV-2 transmission in New York City and the hospitals are geographically distributed around the city to capture different populations. Women were tested uniformly during the study period (there was no sub-sampling). The sample is meant to reflect SARS-CoV-2 prevalence in the population of New York City as a whole to the extent that the prevalence of infection in the women is similar to the prevalence of infection in their communities.
Data collection	Data were recorded by coauthors M Prabhu, D Goffman, YBeilin, R Landau, C Gyamfi-Bannerman, BT Bateman, J Snyder, AS Razavi, D Katz, J Gal, A Bianco, and J Stone at each of the hospital sites upon admission. Data were recorded by computer in Microsoft Excel.
Timing and spatial scale	March 22nd -- May 3rd, 2020 in New York City
Data exclusions	We excluded tests from women with a ZIP code outside of New York City (n = 251) or in Staten Island (n = 14) due to the small sample size from that borough, leaving tests from 1,746 women. These exclusion criteria were established after the data were collected and prior to analyzing the data.
Reproducibility	N/A (this was an observational study)
Randomization	There was no randomization; the study reflects a uniform (complete) sample of women from the four study hospitals.
Blinding	Blinding was not necessary; all women admitted for labor were tested using the same protocol.

Did the study involve field work? ☐ Yes ☒ No

Reporting for specific materials, systems and methods

We require information from authors about some types of materials, experimental systems and methods used in many studies. Here, indicate whether each material, system or method listed is relevant to your study. If you are not sure if a list item applies to your research, read the appropriate section before selecting a response.

Materials & experimental systems

n/a	Involved in the study	
☒	☐	Antibodies
☒	☐	Eukaryotic cell lines
☒	☐	Palaeontology and archaeology
☒	☐	Animals and other organisms
☐	☒	Human research participants
☒	☐	Clinical data
☒	☐	Dual use research of concern

Methods

n/a	Involved in the study	
☒	☐	ChIP-seq
☒	☐	Flow cytometry
☒	☐	MRI-based neuroimaging

Human research participants

Policy information about studies involving human research participants

Population characteristics	Category N %<br>Total 1,746 100<br>Site<br>NYP-CUIMC 385 22.1<br>NYP-LMH 137 7.9<br>NYP-Queens 178 10.2<br>NYP-WCM 290 16.6<br>MSH 428 24.5<br>MSW 328 18.8<br>SARS-CoV-2 test result<br>Positive 244 14.0<br>Negative 1,502 86.0<br>Borough<br>Bronx 309 17.7<br>Brooklyn 386 22.1<br>Manhattan 718 41.1<br>North Queens 275 15.8<br>South Queens 58 3.3<br>Age<br>15-19 21 1.2<br>20-24 167 9.6<br>25-29 346 19.8<br>30-34 588 33.7<br>35-39 470 26.9<br>40-44 139 8.0<br>45-49 13 0.7<br>50-54 2 0.1
Recruitment	There was no recruitment; SARS-CoV-2 screening was universal for all women admitted for delivery.
Ethics oversight	The study was deemed exempt (IRB20-0669) by the Harvard T.H. Chan School of Public Health Internal Review Board on the basis that it was a retrospective secondary analysis of de-identified data

Note that full information on the approval of the study protocol must also be provided in the manuscript.

Clinical data

Policy information about clinical studies

All manuscripts should comply with the ICMJEguidelines for publication of clinical research and a completedCONSORT checklist must be included with all submissions.

Clinical trial registration	n/a
Study protocol	Study protocol is available upon request to the corresponding author, ygrad@hsph.harvard.edu
Data collection	Setting: four NewYork Presbyterian hospital campuses (Columbia University Irving Medical Center/NYP-CUIMC,<br>Weill Cornell Medical Center/NYP-WCM, Lower Manhattan Hospital/NYP-LMH, and Queens Hospital/NYP-Queens), Mount Sinai Hospital (MSH), and Mount Sinai West (MSW) hospital<br>between March 22nd and May 3rd, 2020
Outcomes	Population prevalence of SARS-CoV-2 by borough and correlation with the<br>reduction in daily commuting-style movements into and out of each borough.
